# Synthetic Benzimidazole Opioids: The Emerging Health Challenge for European Drug Users

**DOI:** 10.3389/fpsyt.2022.858234

**Published:** 2022-03-25

**Authors:** Annagiulia Di Trana, Simona Pichini, Roberta Pacifici, Raffaele Giorgetti, Francesco Paolo Busardò

**Affiliations:** ^1^Unit of Forensic Toxicology, Section of Legal Medicine, Department of Excellence of Biomedical Sciences and Public Health, Marche Polytechnic University, Ancona, Italy; ^2^National Centre on Addiction and Doping, National Institute of Health, Rome, Italy

**Keywords:** synthetic benzimidazole opioids, health threat, new synthetic opioids, drugs of abuse, emerging trend

## Introduction

The new psychoactive substances (NPS) phenomenon was initially observed as a response of the illicit market to the international ban on classic drugs of abuse, with the peculiarity of a continuous release of new uncontrolled compounds exerting psychotropic effects which in some cases resulted more potent or longer lasting than those of classic abused substances (1). To date, more than 1,100 NPS have been characterized, mainly new synthetic cathinones and new synthetic cannabinoids, even if the most recent emerging trend has been that of new synthetic opioids (NSOs) (2–4).

In this concern, North America has experienced an unprecedented “opioid crisis” with a notable increase in the rate of opioid-related acute intoxications and fatalities during the last decade. Differently from the past, the new wave of opioid consumption is more related to diversion and misuse of prescription opioids and the emergence of new NSOs characterized by extreme pharmacological potency and toxicity (2). As a result of this opioid epidemic, in 2017, NSOs were involved in 59.8% of opioid-related overdose fatalities in the USA. This trend was confirmed also in 2019, when the synthetic opioid-related fatalities increased in 20 states, ranging between 11.2% (Kentucky) and 95.5% (Colorado) of additional cases (5).With the broadening of the phenomenon toward Europe, in 2020, two-thirds of the global opioid-related fatal intoxications were attributed to NSO misuse, with the healthy-years life expectancy of opioid abusers decreased to 70% (6).

## “Opioid Crisis” From Fentanyl Analogs to Benzimidazole Opioids

In the last few years, the European opioid market has been dominated by fentanyl and its derivatives, originating mainly from illicit manufacture in the Russian Federation area (6). Notably, until 2017 two-thirds of the synthetic opioids detected in the black market were fentanyl analogs (the so-called fentalogues) (4). Between 2017 and 2019, a total of 5,000 kg of fentalogues have been seized in Europe and the number of related intoxications and fatalities reached alarming rates in the same period (7, 8). As an evidence of the increasing popularity of fentanyl and its analogs, heroin has completely been replaced by fentanyl on the black market in Estonia and Finland, being the most marketed synthetic opioid in those countries (4). In response to the uncontrolled rise of this phenomenon, law enforcement agencies and relevant stakeholders (UNODC; EMCDDA; DEA; WHO) joined in a worldwide effort, promoting specific strategies to speed up the risk assessment and scheduling procedure for this NPS class (9, 10). As result of their concerted efforts, in 2020, a sharp decline in the numbers of fentalogue-related deaths was observed (11). Moreover, the COVID-19 pandemic restrictions might have played a role in the reduction of fentanyl analog availability, thanks to the interruption of the NSO supply chain and the improved control of the major hubs for the wholesale trafficking of drugs and precursors, such as land entry and exit points (10, 12).

Starting from 2019, the NSO subclass of benzimidazole opioids (BOs) began to emerge on the illicit drug market to fill the void left by the scheduled fentalogues in the demand for cheaper alternatives to heroin (4).

BOs are synthetic analogs of the controlled μ-opioid receptor agonist etonitazene, discovered in 1956 by the pharmaceutical company CIBA Aktiengesellschaft (Basel, Switzerland) (13). The BO chemical scaffold consists in a benzimidazole core substituted with an N-ethylamine side chain in position 1 and a phenylalkyl chain in position 2 (14). Notwithstanding the structural differences with the classic opioids and the fentanyl analogs, BOs were revealed to have a good affinity for μ-opioid receptor and most of them are characterized by a potency even higher than that of fentanyl (15). The first synthetized BOs differed in their chemical structure for the para substituent on the phenylalkyl moiety: a methoxy group in case of metonitazane, or a clorine atom for clonitazene. Furthermore, a nitro group can be present on the benzimidazole ring in position 5 or 6, such as in case of isotonitazene, metonitazene, or clonitazene and their positional isomers (13, 15). Anyhow, the latter seemed to have a scarce influence on pharmacological activity, as suggested by the potency of some analogs not substituted on the benzimidazole moiety such as isodesnitazene, metodesnitazene, and etodesnitazene (13). Some preliminary *in vitro* studies revealed that BOs might be metabolized in active compounds with potency even higher than that of the precursor, as in the case of N-desethylisotonitazene (15). Although little is still known about BO pharmacology, it is important to highlight that their extreme pharmacological potency was the main reason why the pharmaceutical companies did not invest in their development (16).

The first BO illicitly marketed was etonitazene, detected as a brownish powder in Italy in the late 1960s (13). Thereafter, a series of acute and fatal intoxications related to BOs was reported especially in North America (5), raising concerns from the authorities. More recently, isotonitazene identification in Belgium in 2019 drove the European Monitoring Centre on Drugs and Drug Addiction (EMCDDA) to formally notify it as the first BOs in circulation on illicit market. As a result of this increased concern related to BO consumption, since July 2021, the Toxicology Unit at Imperial College (London, UK) implemented a routine screening analysis to test the blood samples of opioid-related death cases for isotonitazene. As a result, an unexpected number of samples resulted positive, suggesting that, up to that moment, the phenomenon was overlooked (17). Since the first confirmed isotonitazene-related fatality reported in Switzerland in 2019 (18), isotonitazene has been identified in biological samples of about 20 opioid-related fatal cases in Europe (13). Notably, due to the remarkable number of isotonitazene-related deaths reported by third Countries, the EMCDDA issued a risk assessment on this substance (19).

Currently, other analogs such as flunitazene, butonitazene, etodesnitazene, protonitazene, etonitazepyne, and metodesnitazene have been disclosed in the illicit western markets, but related fatal cases or intoxications have not yet been documented (20).

## Conclusion

Although the BOs still embody a smaller class of NSOs, the current European drug market can exert a fertile field for the uncontrolled spread of these substances. The COVID-19 pandemic has contributed to change the NPS market dynamics, due to the intermittent global restrictions that resulted in the shortage of precursors in hot areas and the block of opiates supply chain. On the other hand, the demand for opiates actually increased, pushing the illicit manufacturers to find out always new synthetic and low cost alternatives to satisfy the illegal market requests (12).

Similar to what happened when other NSOs and NPS started to appear on the market, the lack of routine screening analysis able to identify BOS represents a great challenge and important limitation for the prompt identification of the latter (21). Indeed, the recent experience of the Toxicology Unit at Imperial College suggested that BOs were already established in the European territory, but the routine toxicology analysis and strategies based on targeted screening represent a limit in the prompt detection of new unknown substances. Consequently, important public health issues are still underreported. We advocate attention not only on the importance of early identification of illicit drugs to help the risk-assessment procedure but also on the urgency of more effective common preventive strategies to arrest the rise of new harmful substances. Furthermore, the development and implementation of untargeted screening methods (e.g., chromatography–high resolution mass spectrometric methods with software assisted-spectra deconvolution to find out unexpected molecules or the implementation of comprehensive spectra libraries) may represent an important strategy since they allow the structural identification of unexpected substances, consequent identification of those substances in intoxications and fatalities, and the release of risk assessment reports to support the fastest possible inclusion in the banning laws.

As suggested by the most recent data, the opioid crisis is a global issue and hence the spread of new molecules should not be considered as limited to some regions. To this concern, the recent data showed that the international strategies promoted by the UNODC and EMCDDA were demonstrated to be somehow efficacious in contrast to the opioid crisis. However, the recent emergence of the new harmful NPS class suggests that the national and international efforts should be directed to the possible prevention of opioid market changes by strengthening the international network for the drug monitoring.

## Author Contributions

All authors equally contributed to the conceptualization, preparation, and revision of the paper.

## Funding

The opinion was partly funded by the Department of Anti-Drug Policies, Rome, Italy.

## Conflict of Interest

The authors declare that the research was conducted in the absence of any commercial or financial relationships that could be construed as a potential conflict of interest.

## Publisher's Note

All claims expressed in this article are solely those of the authors and do not necessarily represent those of their affiliated organizations, or those of the publisher, the editors and the reviewers. Any product that may be evaluated in this article, or claim that may be made by its manufacturer, is not guaranteed or endorsed by the publisher.
